# Puncture Accuracy of Robot-Assisted CT-Based Punctures in Interventional Radiology: An Ex Vivo Study

**DOI:** 10.3390/diagnostics14131371

**Published:** 2024-06-27

**Authors:** Yannick Scharll, Nenad Radojicic, Gregor Laimer, Peter Schullian, Reto Bale

**Affiliations:** Interventional Oncology-Microinvasive Therapy (SIP), Department of Radiology, Medical University Innsbruck, Anichstr. 35, 6020 Innsbruck, Austria

**Keywords:** imaging, interventional, robotics, robotic surgical procedures, punctures, computed tomography

## Abstract

Objectives: The purpose of this study was to assess the performance of an optically tracked robot for computed-tomography (CT)-guided needle placements in a phantom study. Methods: In total, 240 needle punctures were carried out with the help of an optically tracked robotic device (Micromate) based on CT image datasets at three different slice thicknesses (1, 3, and 5 mm). Conically shaped targets inside a gelatin-filled plexiglass phantom were punctured. The target positioning error between the planned and actual needle trajectory was assessed by measuring the lateral positioning error (ND) between the target and the puncture needle and the Euclidean distance (ED) between the needle tip and target in control CTs. Results: The mean ND and ED for the thinnest CT slice thickness were 1.34 mm (SD ± 0.82) and 2.1 mm (SD ± 0.75), respectively. There was no significant impact of target depth on targeting accuracy for ND (*p* = 0.094) or ED (*p* = 0.187). The mean duration for the planning of one trajectory and for needle positioning were 42 s (SD ± 4) and 64 s (SD ± 7), respectively. Conclusions: In this ex vivo study, the robotic targeting device yielded satisfactory accuracy results at CT slice thicknesses of 1 and 3 mm. This technology may be particularly useful in interventions where the accurate placement of needle-like instruments is required.

## 1. Introduction

Image-guided needle interventions for diagnostic and therapeutic purposes are continuously evolving. Image guidance is accomplished with ultrasound [1], computed tomography (CT) [2], magnetic resonance imaging (MR) [3], and fluoroscopy [4], depending on the target lesion and experience of the physician. Conventional percutaneous CT-guided interventions (e.g., periradicular injections) traditionally rely on the experience of the physician and may involve multiple CT scans that expose the patient to significant radiation [5]. With navigation tools, including robotic guidance systems, these procedures can be simplified and standardized, resulting in better targeting of lesions, reduced procedure time, and improved outcomes [6]. Robot-assisted systems have the ability to hold and move instruments (semi-) automatically with greater precision and accuracy compared to freehand needle positioning [7]. As a result, the number of insertions and readjustments may be reduced, resulting in lower risks of complications, regardless of the clinician’s experience level [8]. Several studies have verified the high targeting accuracy of commercially available robotic systems, both in phantom [9] and animal experiments [10] and in clinical settings [11]. Despite many promising preclinical results, few robotic systems achieved commercialization, and fewer still achieved wide-scale adoption [12].

Throughout the history of robotics, there have been continuous developments to ensure greater accuracy and safety, simultaneously facing the challenge to offer flexibility in choosing the optimal trajectory for the intervention. However, most robotic guidance devices require extensive installation and occupy a lot of space in the operation room [13,14]. Furthermore, multi-probe positioning is often restricted by the end-effector’s limited range of motion. Large robotic devices and/or unwieldy end-effectors make it challenging to avoid colliding with other needles in situations when multiple targets need to be punctured, such as in the radiofrequency ablation of large or multiple liver tumors. These technologies rely heavily on the integration of navigational software and hardware, which must be tailored to physicians in order to minimize variability in operator skill. Inevitably, complex devices are more expensive, so there is a need to balance these conflicting goals.

This work presents a relatively small optically tracked robotic aiming device (Micromate) allowing needle placement based on CT images and promising to circumvent the challenges of multi-angle visual planning. Entry and target points can be directly planned on the system’s console, and the trajectory will be executed without physician calculations of entry-to-target distances and angles. Hence, the aim of this study was to test the accuracy of this novel system in vitro. Additionally, usability, handling time, and drawbacks are discussed.

## 2. Materials and Methods

### 2.1. Phantom

The accuracy analysis was conducted using a rigid plexiglass phantom (220 × 150 × 175 mm) with eight conically shaped aluminum tips mounted on the floor. Their radiopaque tips serve as target points and are spatially distributed at the bottom of the cube at different heights, allowing the operator to puncture at different levels. The target points have a depth up to 13 cm measured perpendicularly from the removable lid. The phantom was filled with gelatin and cooled to 4° Celsius in order to hold the needles in place for the CT evaluation and mimic the consistency of an internal organ, such as the liver. The same phantom had previously been used to analyze the accuracy of other guidance systems [15,16,17,18,19,20].

### 2.2. Micromate Navigation System

**Figure 1:**
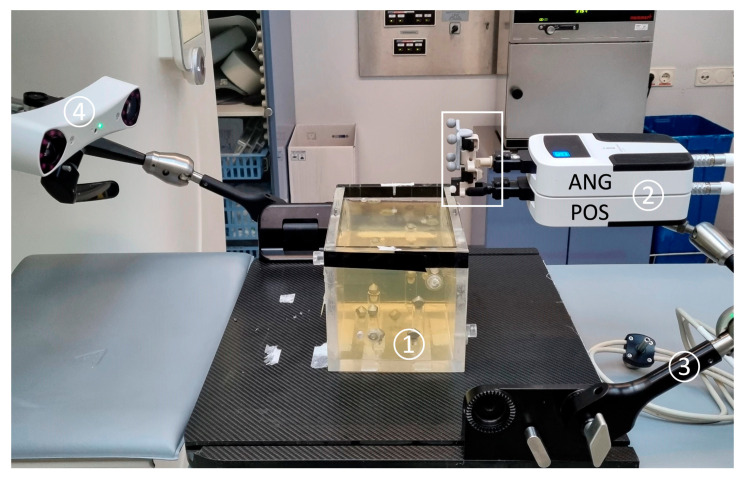
The gelatin-filled phantom (1) glued to the black carbon plate. The RPU (2) can be manually prepositioned by a 7DOF multifunctional arm (3) mounted on the side rail of the carbon plate. The optical camera (4) detects the 4 passive infrared-light-reflecting markers clamped onto the end-effector (white frame). The camera can emit a laser beam to facilitate alignment with the markers.

**Figure 2:**
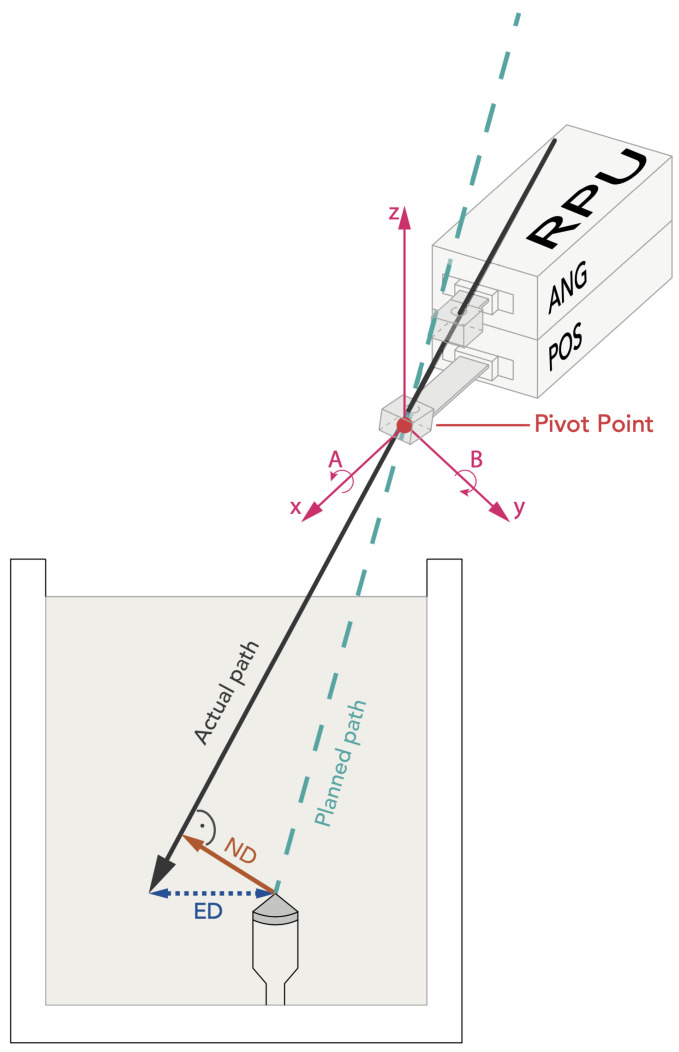
Schematic illustration of the RPU after robot-assisted alignment. The puncture needle is inserted based on a double oblique plan. The reflective markers are not depicted for clarity purposes. The pivot point of the guidance sheath is superimposed with the virtual elongation of the planned trajectory (*X*- and *Y*-axes), and the angulation in A and B allows for the alignment of the double oblique needle trajectories.

The experimental setup is shown in Figure 1. The Micromate robotic guidance system (iSYS Medizintechnik GmbH, Kitzbühel, Austria) assembly consists of a robot needle positioning unit (RPU) with four degrees of freedom (DOF) that is mounted on a passive holding arm with seven degrees of freedom. The RPU is divided into two modules: a lower part and an upper part (Figure 2). The lower 2DOF positioning part (POS) allows the end-effector to move transversally, being restricted to a field of 40 mm by 40 mm (X × Y). The upper part (ANG) serves for the 2DOF angulation of the needle up to ±32° and ensures rotation around the *X*- and *Y*-axes. In the *Z*-axis, the end-effector cannot be moved other than with a hand.

System components include a treatment planning workstation that supports 3D DICOM CT images and a camera (spryTrack 180, Atracsys LLC, Puidoux, Switzerland) for optical tracking of four passive infrared-light-reflecting markers attached to the needle holder. Four radiopaque titanium spheres integrated in the end-effector serve as fiducial registration markers to automatically register the iSYS system using the planning scan.

### 2.3. Experimental Setup

**Figure 3:**
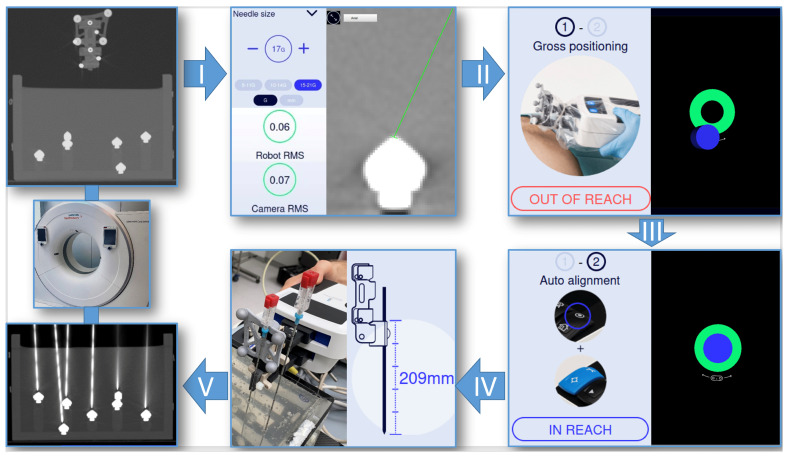
Workflow of the Micromate system. First, a planning CT scan is performed including the target area as well as the spherical radiopaque balls and reflective markers for automated registration. I: After DICOM image transfer, sequential planning of 6 trajectories is possible after selecting the needle size, in this case 17 G. The target and entry point are selected on the workstation’s touch screen. Root mean square errors are given to monitor the accuracy. II: Once the trajectory planning is confirmed, positional information is converted to robot coordinates and sent to the controller. The passive 7DOF multifunctional holding arm mounted to the carbon plate is used for manual gross positioning. A blue cylinder illustrating the virtual alignment of the end-effector is grossly superimposed with a green circle indicating the target area. III: When the robotic device is in reach, auto-alignment by the robot can be executed. IV: The correct needle length is shown on the screen. The needle can then be inserted manually until the marked depth is reached. V: Maximum-intensity projection of the control CT. In order to evaluate accuracy, the post-procedure CT scan is transferred to the Treon workstation via the hospital’s intranet.

A Siemens Somatom Sensation Open CT system (Siemens Healthineers, Erlangen, Germany) was used to acquire 1, 3, and 5 mm planning CT datasets (100 kV, 50 mAs, detector configuration 32 × 0.6 mm, FOV of 240 mm) of the gelatin-filled phantom. For automatic registration, the field of view must include the target area as well as the fiducial markers (Figure 3). The reformatted DICOM images were then transferred to the workstation via USB flash drive. The system automatically performs registration and displays the root mean square error (RMS), which is an indication of the registration accuracy. In addition, the RMS of the camera and the reflective markers is an indication of the optical tracking accuracy. Thereafter, six double oblique trajectories were planned on the basis of multiplanar reformatted images by defining the target (tip of the cone) and an arbitrary entry point. Upon completion of the planning process, positional information was transformed into robot coordinates allowing for robot-assisted needle placement. After automatic alignment of the RPU, the insertion depth was displayed and marked on a 17 G needle with a pen. The needle was inserted through the needle holder of the robotic arm to the preplanned depth.

### 2.4. Evaluation

Post-procedural control CT scans with 1 mm slice thickness (120 kV, 100 mAs, detector configuration 32 × 0.6 mm, slice thickness 1 mm, reconstruction interval 1 mm, FOV of 240 mm) were transferred to the “Mach Cranial” software of the StealthStation Treon (Medtronic, Louisville, KY, USA) for accuracy evaluation. Therefore, the coordinates (x, y, and z) of the planned target, the needle tip, and a random point on the needle near the gel entry point were extracted. Euclidean distances (EDs) and normal distances (NDs) were calculated. The ED is defined as the distance between the actual needle tip position and the target point. The normal distance is defined as the shortest possible distance between the target point and the needle (Figure 2). A detailed explanation of the definitions and formulas can be found in the following source [21]. All statistical analyses were performed using SPSS Version 22 (SPSS Inc., Chicago, IL, USA). The distribution of error measurements was graphically checked with histograms and assessed with the Kolmogorov–Smirnov Test. Mean errors, standard deviations, and ranges were calculated. In order to evaluate the difference between slice thicknesses, an independent *t*-test was conducted. Statistical significance was determined by a *p*-value of <0.05.

## 3. Results

In order to minimize the impact of a learning curve, 80 punctures were performed for practice purposes. A total of 240 CT-guided punctures were then performed and evaluated.

### 3.1. Accuracy

**Figure 4:**
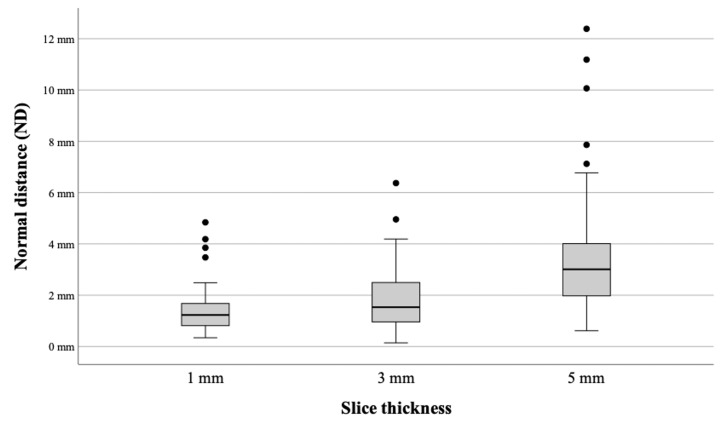
Comparison of the Micromate accuracy across all slice thicknesses.

The mean ED from the needle tip to the target for 1 mm, 3 mm, and 5 mm was 2.1 mm (SD ± 0.75), 2.35 mm (SD ± 1.13), and 3.93 mm (SD ± 2.78), respectively. The accomplished mean ND from the needle axis to the target for the 1 mm, 3 mm, and 5 mm slice thicknesses was 1.34 mm (SD ± 0.82), 1.8 mm (SD ± 1.12), and 3.46 mm (SD ± 2.13), respectively (Figure 4). The minimum and maximum deviations, as well as the comparison with the optically tracked predecessor system iSYS-1 in combination with the Medtronic Stealth Station S7 are provided in Table 1. For both experiments, the same phantom and CT slice thicknesses were used.

The Kolmogorov–Smirnov test showed a normal distribution. Using the Student’s *t*-test, a significant difference in accuracy (ED and ND) could be observed between all slice thicknesses except for the ED of 1 mm versus 3 mm (*p* = 0.095).

ED: 1 mm vs. 3 mm (*p* = 0.095), 1 mm vs. 5 mm (*p* < 0.01), and 3 mm vs. 5 mm (*p* < 0.01)
ND: 1 mm vs. 3 mm (*p* = 0.02), 1 mm vs. 5 mm (*p* < 0.01), and 3 mm vs. 5 mm (*p* < 0.01)

The target depth was determined by measuring the distance between the needle entry point on the gel surface and the target point. All 240 punctures were performed with a mean depth of 67.45 ± 15.47 mm (range 31–104 mm). We divided the punctures into two groups (group 1: target depth < 65 mm; group 2: target depth ≥ 65 mm). According to the *t*-test, the target depth did not significantly affect targeting accuracy in either of the two samples (*p* = 0.094 for ND and 0.187 for ED).

### 3.2. Procedural Time

**Figure 5:**
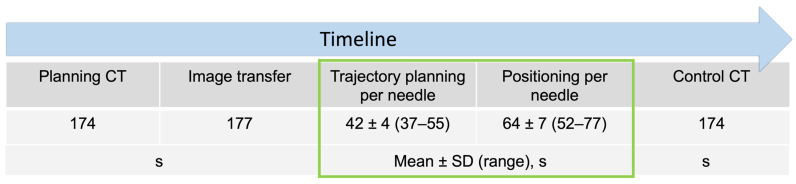
An illustration of procedural events and their timeframe in seconds (s). The given procedural durations refer to a single needle.

The mean duration of the total procedure including image acquisition, image transfer, trajectory planning, and sequential positioning of six needles was 16.5 min. A detailed description is given in Figure 5.

## 4. Discussion

The objective of this phantom study was to examine the performance of a newly available stereotactic robotic guidance system with optical tracking intended for different types of surgical and radiological interventions. When implementing a navigation system for puncture guidance, accuracy and safety are crucial factors. By improving puncture accuracy, intervention success rates may increase and complication rates may be reduced.

### 4.1. Targeting Accuracy

In clinical practice, the achieved mean ED of 2.1 ± 0.7 mm between the needle tip and the target would be acceptable. In fact, the target lesions must be at least 10 mm in diameter if we are planning to take a biopsy. The correction of depth deviations requires deeper needle insertions or retractions. Since the lateral deviation necessarily requires manual re-angulation or reinsertion, the ND (lateral positional error) is of greater importance. A mean ND of 1.3 ± 0.8 mm at a 1 mm CT slice thickness is comparable to results previously reported in the literature using alternative CT-based navigation techniques [7,16,22]. Due to differences in study endpoints, insertion angles, target depths, phantom materials, and needle types and gauges, comparisons between studies are limited. In a gel phantom study, Pollock et al. [22] compared the performance of one of the earliest CT-compatible robotic systems with a computer-assisted optical navigation system. With the AcuBot robot, a mean ED of 1.2 mm (range 0.4–2.8 mm) was achieved. In the CT workflow, registration involved manually aligning the needle with the laser marks of the CT scanner, whereas in our study, registration was automatic. In a recent study, Spenkelink et al. [7] assessed the performance of another robot (ANT-C system) for CT-guided needle positioning procedures using an abdominal phantom. With the robot, needle positioning was more accurate and successful than with freehand needle positioning. The robot was able to place the needle in the target area with a mean lateral deviation (ND) of 2.3 ± 1.4 mm. In contrast to the flexibility of the Micromate, the larger ANT-C must be mounted on a CT-table and requires four CT scans for registration of the robot and image coordinate system. In an accuracy study, de Baere et al. investigated the optically tracked robot EPIONE, which is automatically registered by using a reference frame. A respiratory-monitoring module was integrated and repeatability tests were performed during expiration in order to increase patient safety. Radiopaque markers surgically inserted into the kidneys of two pigs were targeted by 17 G needles from different trajectories. The study was limited to eight punctures with a mean ND of 2.3 ± 1.2 mm and a median procedural time (from starting the system to the final control CT) of 21 min. Such large and sophisticated robotic systems are cumbersome and expensive, yet the optically tracked EPIONE offers high flexibility covering a large treatment volume. Levy et al. assessed the accuracy of a patient-mounted robot during 32 percutaneous abdominal and pelvic biopsies. The overall accuracy was 1.6 ± 1.5 mm (mean ND ± SD). The compact robotic system (XACT ACE) provides automatic needle insertion and automated re-adjustment of the needle trajectory according to specially selected checkpoints. Yet, it is important to mention the very limited range of motion resulting in a small treatment volume, which may require repositioning and a repeat CT scan. In contrast, the optically tracked Micromate has no limitations in selecting the entry point and angulation of the needle path as long as the reflective markers are detected.

Prior to this study, the same phantom was used for a variety of accuracy studies on robotic devices [15,16,18], patient-mounted navigation [15], patient-specific template-based guidance [20], optical navigation and laser guidance in combination with an aiming device [17,18], as well as electromagnetic navigation systems [19]. Accuracy results are summarized in Table 1. The accuracy level achieved by Micromate is comparable to that of the other robots. For the Innomotion, Maxio, and Micromate robots, the ND ranges from 1.3 to 1.4 mm at the thinnest CT slice thickness. One exception is the optically tracked iSYS-1, which achieves submillimetric accuracy. From a clinical point of view, all the mentioned robotic devices are within acceptable error ranges of currently used interventional techniques (e.g., biopsy, tumor ablation, etc.).

With the 5 mm slice thickness we had some outliers, with an ED and ND greater than 10 mm. We conclude that this might have been due to registration errors of the four spherical titanium fiducial registration markers having a diameter of 5 mm, as they were more difficult to register on coarser CT slice thicknesses. Anyway, a slice thickness of >3 mm has limited clinical value due to difficulties in defining the target. To avoid losing the registration, neither the phantom nor the camera must be moved. As a preventative measure, the robot, camera, and phantom were all fixed to the same plate.

### 4.2. Time Efforts

In a mean time of 16.5 min from the starting time of the CT scan to puncture completion, six 17 G needles could be sequentially positioned. The set up time was not included. Yet, installation was simple due to the small size and weight of the robot, the light carbon plate, and the option to mount the planning monitor on a cart. In the patient, additional draping has to be considered. Nevertheless, the robot instantly processes the 3D data set uploaded via USB flash drive, and the planning process is intuitive, allowing the user to select the entry and target points quickly.

In terms of future clinical applications, the Micromate system exhibits a number of promising features. Automatic registration has been successful in all experiments. For automatic registration of the robot and image coordinate system, a single planning CT scan over the region of interest including the end-effector is sufficient. Neither additional sensors nor additional patient markers are required. This in turn reduces procedural time requirements, especially when compared to conventional optical registration procedures based on the manual definition of markers [16]. Compared to the quite unwieldy end-effector of the Innomotion and the Maxio, the small size of the Micromate’s control unit increases flexibility in selecting different entry points. As opposed to floor-mounted systems, it can be used in virtually any type of CT scanner. With the robot mounted on the CT tabletop, needles may be positioned inside the CT gantry. Due to the radiolucent end-effector, needles may be advanced using CT fluoroscopy [23]. Currently, Micromate software supports three workflows. In addition to the optical workflow presented in this study, there is the option to use the CT workflow without a camera. There is one significant disadvantage to this workflow, similar to the previously mentioned XACT robot: the end-effector must be manually prepositioned within an area of 40 × 40 mm near the desired entry point. In the event of a misjudgment of the target location, the robot must be repositioned, which results in a repeat CT scan. The third workflow enables robot-assisted guidance only relying on one pair of 2D C-arm images. This approach can be particularly useful for orthopedic purposes.

Movements of the target region, especially those caused by breathing, cannot be compensated for by the system. In order to overcome this limitation, images and interventions must be performed in the same breathing mode by disconnecting the endotracheal tube in anaesthetized patients [24]. Furthermore, the patient’s breathing can be tracked in real time and quantified by using a respiratory-monitoring module [25] or the patients can be coached to respond to given breathing commands using a strain belt [26]. It is especially dangerous for the patient to move while the needle is already inserted in the body, even though the needle can be easily released from the needle holder. Therefore, patient immobilization using a vacuum mattress, for example, is highly recommended to ensure comfort and stable patient positioning. In addition, it must also be ensured that the camera is mounted at a sufficient distance from the end-effector to allow for error-free tracking of the markers [27].

In conclusion, our results of a phantom puncture series show that accurate CT-guided punctures can be achieved using the Micromate, a novel optically tracked robotic system. Physicians with less experience might find it easier to perform challenging multi-angle punctures this way. The compact system design and automatic optical registration simplify the application. The highest accuracy was achieved with a 1 mm CT slice thickness. However, there are still a number of drawbacks, such as lack of ability to compensate for respiratory movement. In addition, it is crucial to balance efficacy with cost implications. Further studies are desirable to evaluate the potential clinical use in various stereotactic interventions.

## Figures and Tables

**Table 1:** Accuracy of the Micromate compared to previously reported results of our group using the same phantom.

Slice Thickness		Micromate	iSYS-1 S7	Innomotion	ArciNav	Stealth Station Treon	AxiEM	PercuNav	PCS	Slice Thickness	Maxio	SimpliCT +Atlas
			Scharll et al. [16]	Stoffner et al. [18]	Venturi et al. [20]	Stoffner et al. [18]	Putzer et al. [19]	Putzer et al. [19]	Scharll et al. [15]		Scharll et al. [15]	Scharll et al. [17]
**1 mm**										**1.25 mm**		
	ED mean (mm)	2.1	1.6	1.7	2.5	1.9	3.9	4.4	4.1		1.5	2.8
	ED min. (mm)	0.9	0.8	0.5	0.7	0	1.1	2.4	0		0	1.1
	ED max. (mm)	5.3	2.5	3.3	4.6	4.8	15.8	8.7	8.0		3.3	5.4
	ED SD (mm)	0.7	0.4	0.8	0.6	0.9	2.3	1.3	1.7		0.9	0.9
	ND mean (mm)	1.3	0.9	1.4	1.4	1.6	3.3	3.8	3.8		1.3	1.8
	ND min. (mm)	0.3	0.2	0.2	1.2	0	0.3	1.3	0		0	0.2
	ND max. (mm)	4.8	2.2	3.1	2.6	4.6	9.9	8.7	7.8		3.2	5.1
	ND SD (mm)	0.8	0.4	0.8	0.7	0.9	1.5	1.6	1.8		0.8	0.9
**3 mm**										**2.5 mm**		
	ED mean (mm)	2.4	2.0	1.9	2.8	2.2	3.7	4.3	4.6		1.6	3.4
	ED min. (mm)	0.4	0.7	1.0	0.5	0.6	1.1	2.1	0		0	2.0
	ED max. (mm)	8.5	3.0	3.9	5.3	5.5	10.3	8.0	9.9		6.7	5.7
	ED SD (mm)	1.1	0.5	0.7	1.0	1.1	2.1	1.3	2.3		1.2	0.8
	ND mean (mm)	1.8	0.9	1.6	2.0	1.8	3.2	3.8	4.4		1.3	2.1
	ND min. (mm)	0.1	0.2	0.2	0.2	0.1	0.4	1.0	0		0	0
	ND max. (mm)	6.4	1.7	3.7	5.2	5.1	8.4	7.7	9.3		3.4	5.4
	ND SD (mm)	1.1	0.5	0.7	1.1	1.2	1.5	1.4	2.3		0.8	1.1
**5 mm**										**5 mm**		
	ED mean (mm)	3.9	1.8	2.3	No data	2.7	4.8	4.5	4.6		1.7	3.7
	ED min. (mm)	0.6	0.6	0.7	No data	0.3	1.3	2.1	0		0	2.3
	ED max. (mm)	15.4	2.9	5.2	No data	6.8	10.5	9.2	8.9		7.1	7.6
	ED SD (mm)	2.8	0.5	0.9	No data	1.2	2.1	1.6	2.1		1.2	1.1
	ND mean (mm)	3.4	0.7	2.0	No data	2.5	3.9	3.8	4.4		1.4	1.9
	ND min. (mm)	0.6	0.1	0.5	No data	0.1	0.1	0.5	0		0	0.1
	ND max. (mm)	12.4	2.2	5.2	No data	6.6	8.8	8.1	8.8		7.0	7.5
	ND SD (mm)	2.1	0.5	1.0	No data	1.2	1.7	1.7	2.1		1.0	1.4

## Data Availability

The data that support the findings of this study are available from the corresponding author, BR, upon reasonable request.
